# Decision-Making Within Forensic Psychiatric Investigations: The Use of Various Information Sources by Different Expert Groups to Reach Conclusions on Legal Insanity

**DOI:** 10.3389/fpsyt.2022.822519

**Published:** 2022-04-14

**Authors:** Lizel Göranson, Olof Svensson, Peter Andiné, Sara Bromander, Ann-Sophie Lindqvist Bagge, Malin Hildebrand Karlén

**Affiliations:** ^1^Department of Psychology, University of Gothenburg, Gothenburg, Sweden; ^2^Centre for Ethics, Law and Mental Health, Department of Psychiatry and Neurochemistry, Institute of Neuroscience and Physiology, Sahlgrenska Academy, University of Gothenburg, Gothenburg, Sweden; ^3^Department of Forensic Psychiatry, Swedish National Board of Forensic Medicine, Gothenburg, Sweden; ^4^Forensic Psychiatric Clinic, Sahlgrenska University Hospital, Gothenburg, Sweden

**Keywords:** decision-making, forensic psychiatric investigation, psychiatric assessment, legal insanity, expert evaluation, court order

## Abstract

**Background:**

Which type of information experts use to make decisions regarding legal insanity within forensic psychiatric investigations (FPI) is relatively unknown, both in general and when considering variations due to case context. It is important to explore this area to be able to counteract the effects of various kinds of cognitive bias.

**Method:**

The aim was to explore whether FPI expert groups differed regarding case-specific as well as general use of information types required to make decisions on severe mental disorder (SMD). Three FPI case vignettes were presented to three professional groups involved in FPIs in Sweden (*n* = 41): forensic psychiatrists (*n* = 15), psychologists (*n* = 15), and social workers (*n* = 11). The participants reported which types of information they required to reach conclusions regarding SMD in each case. They also reported which types of information they had used within general FPI praxis during the previous year and the information types’ perceived usefulness.

**Results:**

The expert groups differed somewhat regarding what type of information they required for the cases (e.g., results from cognitive testing), but some information was required in all cases (e.g., client’s self-report). Regarding the preliminary assessment of SMD in the three cases, minor differences were found. Within the general FPI praxis, experts reported using several information types, while the general perceived usefulness of these sources varied.

**Discussion:**

The professional groups relied partly on a “core” of information sources, but some case-specific adaptations were found. The professional groups’ inclination to suspect SMD also varied somewhat. This indicates a need to explore the potential consequences of these similarities and differences.

## Introduction

The decision-making processes within forensic psychiatric investigations (FPI) are highly complex and vary between cases, and experts may even disagree on its conclusion (1). In order for the conclusions reached to be valid, FPIs need to be performed according to the best available current evidence on complex decision-making (2, 3). Since empirical data regarding FPI decision-making processes is largely lacking, it is important to begin with outlining what kind of information these decision-making processes are based on and relate this to the confirmed risk of the FPI experts’ decision-making processes being affected by different biases (i.e., risks resulting in unequal legal treatment) (4). The present study explored what types of information sources were generally used in FPI praxis in Sweden and tested their application within various case contexts, illustrating the current information basis for the FPI’s decision-making processes and also discussing specific high-risk areas for bias.

In research on decision-making in general, and complex decision-making in forensic investigations in particular, the dual-process theory (5) has been used (2). The dual-process theory has, since its initial conception [e.g., (6)], undergone changes (3, 7), but the central dividing of type 1 processing from type 2 processing remains. Decisions based on the more automatic type 1 processing are made quickly and virtually effortless, but when more complex problems emerge, type 1 processing should be replaced by the analytical type 2 processing. Type 2 processing requires considerably more focus and nuanced evaluation of information to solve the problem as appropriately as possible based on available facts. These two processes enable individuals to solve problems either fast/automatically or slow/analytically, but type 1 processing is the “default,” and this kind of processing increases the risk of unwanted bias effects on decisions (8). Within FPI decision-making, this could be detrimental to the integrity of the conclusions due to rule of law (i.e., everybody is equal before the law and only relevant factors should impact legal decisions).

To provide reliable and accurate conclusions to the court, FPI experts need to make skilled observations and conclusions with as little influence of bias as possible (9). The term “bias” is often used to describe an individual’s emotional involvement in a situation, but it can also be used to describe systematic cognitive errors that a person makes (10). It is very difficult to make decisions free from bias (both cognitive and emotional) (11, 12), and professionals are not immune. In fact, it has been suggested that experts could be even more vulnerable to bias if they trust too much on their own experience, which can decrease their effort to conduct a thorough examination of all available facts in a case (9, 13). Even when motivated to be unbiased, experts in forensic decision-making nevertheless seem to be susceptible. Since FPIs include making complex decisions, there is reason to believe that experts could be susceptible to various forms of bias (4). One such bias is the “bias blind spot,” which refers to the common human tendency of recognizing bias in other individuals but fails to do this in oneself (14). This tendency to underestimate one’s own bias compared to their colleagues has been identified in studies on forensic mental health professionals (10, 15). Neal and Brodsky (10) argued that such a bias induce overconfidence in the expert’s own judgment which could lead to risky decision-making, including rejecting other professionals’ divergent ideas which, if considered, potentially could have reduced the impact of bias and improved the decision validity.

Regarding bias in forensic sciences, Dror (16) presented a theoretical model outlining seven sources of bias (and underlying causes) that can affect experts within forensic sciences. The most basic kinds stem from human “wiring” (i.e., “cognitive architecture and the brain”, “training and motivation”), others come from the environment, culture, and experience (i.e., “organizational factors,” “base rate expectations”), and others are from the specific case context (i.e., “irrelevant case information,” “reference materials,” “case evidence”). Additional research confirms professional training as a factor biasing forensic decision-making (17). To minimize bias in forensic work, these sources of bias must be understood, and through understanding how they occur, counter-measures can be developed (16, 18). In the hierarchy of expert performance (HEP), the “observation” and the “conclusion” elements in expert decision-making are distinguished, designating when bias can cause different experts to reach different conclusions based on the same information. Within this model, Dror (16) discusses, among other things, the concept of “biasability,” which includes the potential effect of irrelevant contextual information and other biases that may influence decisions. By using HEP, Dror (18) argues that research studies can be organized and conceptualized and a clear theoretical framework can be obtained; at the same time, there will be focus on reliability and biasability issues that cut across expert domains. Based on this model, it could be assumed that, by using more and more varied information sources, the impact of such different kinds of bias on decision-making could be diminished and increase the chance of type 2 processing to permeate the FPI decision-making process within various professional groups working with FPIs. Related to this, the importance of multi-method assessment has also been argued for, within research on psychological assessment praxis, gathering information from different sources and perspectives is a vital part of state-of-the-art psychological assessments today (19). This approach decreases the risk of bias affecting decisions [e.g., (20)] as well as increases the accuracy of decisions especially when standardized methods are used to inform the clinical assessment (21).

The forensic psychiatric investigations within the Swedish justice system differs in some respects to those of other countries. A central focus of the FPI is the legal concept of severe mental disorder (SMD), a psycho-legal term used in Sweden to differentiate between offenders who are normally sentenced to forensic psychiatric care rather than imprisonment (22, 23). When experts come to opposing conclusions regarding SMD, it may lead to diminished public confidence in the reliability of these assessments as well as in forensic psychiatric praxis in general (24). According to the legislative bill preceding the criminal code (25), the construct of SMD primarily encompasses psychotic states (e.g., delusions, thought disorders) or equivalent mental states, including severe personality disorders (e.g., with severe obsessive–compulsive traits and/or severe impulsive breakthroughs) and certain neuropsychiatric disabilities, which should be equivalent in degree to a psychotic state with impaired or loss of reality orientation [i.e., related to the concept “legal insanity”; see Svennerlind (23) and Bennet and Radovic (26) for a discussion]. In 2020, 529 FPIs were conducted in Sweden (men: *n* = 462), and 57% of the FPI clients were considered to have an SMD at the time of the crime (27). These investigations are performed on behalf of the court by the National Board of Forensic Medicine, Department of Forensic Psychiatry (DFP). An FPI is based on comprehensive, multi-professional assessments conducted within a team setting, where the court questions whether the person’s mental state corresponds to a SMD at (a) the time of the crime and (b) at the time of the FPI (27). For a suspect in custody, an FPI lasts for a maximum of 4 weeks, during which the client is taken from police custody to stay at the ward at the DFP.

While the FPI team structure in Sweden includes four professional groups—forensic social workers, forensic psychologists, forensic psychiatrists, and nursing staff—England and Wales only include two physicians, of which one must be a psychiatrist who contributes to the assessment (28). In the United States, forensic mental health experts (including psychiatrists and psychologists) conduct assessments (and can be retained by one side or another in a case to testify in court) (4, 29), while in Norway two general specialists in psychiatry or one specialist in clinical psychology and one psychiatrist are appointed to make the assessment (30). In other parts of the world, such as Indonesia, it is also physicians/psychiatrists who conduct this kind of forensic assessments (1). Although Holland requires the participation of more than one professional group apart from psychiatrists [most often psychologists; see Messina et al. (31)] within assessments, Sweden’s routine inclusion of four different expert groups in FPIs is unique in the European Union (28) and, to the best of our knowledge, in the world. In Sweden, each profession not only conducts their own assessment and writes a report submitted to the court but they also work together with a representative from all other professional groups in a team setting. As a standard, three team conferences are held during the course of an FPI. However, only the first three groups give an opinion regarding SMD from their different professional perspectives. The forensic psychiatrist, who has the overall responsibility for the FPI, gives the final recommendation regarding SMD (yes/no) to the court based on the different reports. Hence, despite the differences between Sweden and the other countries described above, Sweden is similar to many other countries regarding which professional group has the overall responsibility for the SMD decision delivered to the court. The assessment of SMD shall therefore be based on the perspectives and methods used by the respective professional groups, and the documents guiding each group’s FPI praxis within DFP are described below.

The forensic social worker is responsible for providing documentation regarding psychosocial functioning and illustrating the person’s life history. They formulate how previous experiences may have affected the person later in life and investigate the person’s level of psychosocial functioning. This includes an investigation of relevant factors in the person’s childhood, adolescence, and current situation [e.g., employment, substance abuse, criminal lifestyle, and social aspects of mental disorders (32)].

The forensic psychologist focuses on various aspects of psychological dysfunction and on psychiatric disorders, considering also various factors that could affect cognitive functioning (e.g., substance use, traumatic brain injury). Various psychological tests and other assessment methods are often used to illustrate the clients’ cognitive and personality functioning in a standardized manner. This information is then related to the person’s psychological functioning at the time of the crime and of the FPI (33).

The forensic psychiatrist is responsible for the FPI as a whole and writes two reports; first, a medical–psychiatric assessment and, second, the final summary FPI report (34, 35). Examples of aspects to consider in the medical–psychiatric assessment from a psychiatric perspective are familial heredity (e.g., mental illness, somatic diseases), psychiatric and physical medical history/current state (i.e., previous psychiatric diagnoses, epilepsy, somatic injuries), substance use/abuse, the client’s behavior during the FPI (including the attitude toward their crime), and how such aspects could have affected the client’s mental state at the time of the crime and FPI. The final FPI report is based on the above-mentioned medical–psychiatric assessment, the other professional group’s reports, and a report from the nursing staff at the FPI ward (e.g., clinical impressions and behavior observations during the client’s stay).

In sum, a vast amount of information can be acquired when conducting an FPI, and by having three professional groups considering the information both separately in their respective reports and together during the team meetings, an FPI is indeed a highly complex decision-making process that, as such, can be vulnerable to various kinds of bias. As previously mentioned, there has been some international research illustrating the case and/or assessment context’s influence on the expert’s conclusions regarding (a) legal insanity [see (36, 37)] and (b) SMD (i.e., the approximate equivalent to legal insanity in Sweden) (38, 39). However, to the best of our knowledge, no previous research has explored what kind of information forms the basis for these decisions, thus shaping the decision-making process of FPIs in Sweden either in a general manner or when the case context is varied.

The purpose of the present study was to explore whether FPI experts from different professional groups (i.e., forensic psychiatrist, forensic psychologist, forensic social worker) differed regarding how many information sources and which types they would require to make decisions on SMD in different types of cases (see part 1). The aim was also to explore the use of information in general FPI praxis, focusing on which types of information they had based their FPI decisions on during the past year and how useful these different types of information had been (see part 2 and part 3). The research questions were the following:

Part 1

1. Do the professions differ regarding how many information sources they required to conduct their FPI assessment in three different case contexts (here case vignettes)?
2. Do the FPI experts adapt their type of required information to these three case contexts?
3. Do the professional groups differ in their conclusion regarding SMD at the time of the (a) crime and (b) FPI within these case contexts?

Part 2 and Part 3

4. Do the professional groups differ regarding (a) what type and (b) how many information sources they have used during the past year and also (c) how helpful they perceive these different information sources to have been?

## Materials and Methods

This study was part of the research project “Decision-making in forensic psychiatric investigations: theory and practice,” with the purpose to illustrate the decision-making process within FPIs at the National Board of Forensic Medicine in Sweden. The project’s data was collected during November and December 2019. The project was approved by the Swedish Ethical review authority (Dnr: 940-16).

### Participants

A list of all experts currently working with FPIs at the DFP in Sweden was compiled and, *via* e-mail, informed and invited to participate (*n* = 66, one participant was excluded due to long-term sick leave): forensic psychiatrists (*n* = 27, of which seven were residents in forensic psychiatry, specialists in general psychiatry), forensic social workers (*n* = 19), and forensic psychologists (*n* = 20). If the invitee agreed to participate, they were instructed to respond to the e-mail, sign the attached informed consent form, and choose among the specified time slots for participation. After two subsequent reminders *via* e-mail, 33 experts, in total, expressed their interest. However, since some more experts who had expressed their interest to participate were not available on the suggested dates (e.g., short-term sick leave, holiday), additional time slots were suggested, resulting in the participation of eight additional experts. The final sample (*n* = 41) consisted of 15 forensic psychiatrists (with three residents), 15 forensic psychologists, and 11 forensic social workers. The participation rate from the initial invitation was 62%.

### Instrument and Measures

Three case vignettes were used to gather both quantitative data (i.e., concerning the use of certain information sources and the conclusions noted in a response form) and qualitative data (i.e., answers to open-ended questions generating written responses, not presented here). Before reading the vignettes and answering its response form, a semi-structured interview was conducted with the participants [see Svensson et al. (40), for more information].

#### The Response Form

**FIGURE 1:**
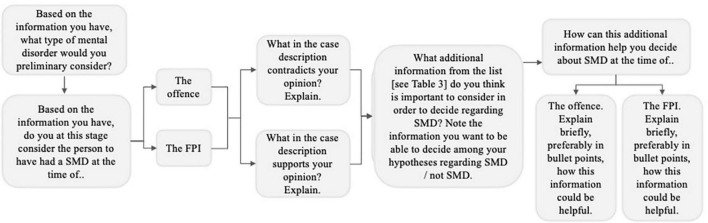
Flowchart representing the sequence of the questions in the case vignette part (part 1) of the response form.

The response form and case vignettes were created by clinicians both within the DFP (MK, PA, and OS) and in general mental health practice (ASLB). A non-clinical researcher also participated (SR) in order for the material to be suitable tools for answering the research questions. The response form (see Figure 1 for overview) was pilot-tested by one representative from each profession with whom the list of information sources was also discussed to see if any source needed to be changed or added. On the first page of the response form, the participants were given a brief introduction to the three-part structure of the form, and background variables were also collected regarding (1) how many FPIs they had participated in and (2) their profession (i.e., forensic psychologist, forensic social worker, or forensic psychiatrist). The response form consisted of three parts. In part 1 of the response form, the participants read the three case vignettes describing the clients undergoing an FPI, with varied psychiatric profile and context behavior during the FPI, and although the crime was the same (aggravated assault), its context also differed (e.g., victim, setting). Each case vignette was created not only with a particular ideal type in mind (here a type of case that was considered to be fairly common in the FPI context and also related in different manners to the nuances of the SMD construct) but also somewhat ambiguous and not presented as clear cut regarding either the psychiatric problem profile or SMD. The case vignettes are summarized in the discussion below.

#### Vignette 1

A 23-year-old man was charged with aggravated assault on a university classmate. He was described during the FPI to be reserved and suspicious, having told the FPI team that his classmates had laughed behind his back (cited as one reason for the assault). He had confessed and said that he had been drinking a couple of bottles of beer before the assault. He tells the FPI team that he has had a normal childhood, had friends, and had no current or historical alcohol or drug abuse but that he has been lately drinking more beer than usual. It has been hard for him to cope with school which had led to sleep difficulties, increased stress, and irregular eating routines. His life data (i.e., principally record-based information) indicated no contact with a child psychiatrist but that he later, in life, had sought psychiatric care for depression, anxiety, and increasing isolation (but never medicated). His childhood seemed to have been happy, and his relatives stated that no behavior problems were observed during his youth. He had passing grades during primary school and high school. At 20 years old, he moved to a new city for university studies, where he had problems finding new friends. In intellectual testing, his overall IQ level was within the normal range, but his results regarding processing speed were within the lower part of the normal range. Taken together, this vignette was meant to reflect a person with possible psychosis by highlighting different key characteristics, such as suspiciousness/paranoia, the nature of violence, and distorted perception of reality. However, the subtlety of symptoms and potential soundness of his interpretation of his classmates’ behavior (where information was lacking) could be arguments against SMD in this case.

#### Vignette 2

A 29-year-old man was charged with aggravated assault on an acquaintance. During the FPI, he greeted the team politely, quickly took command of the situation, and appeared accommodating and carefree upon social interaction. However, it soon became clear that he could not handle being contradicted, and he asked, in a threatening manner, if there was any other investigator who might understand his situation better. Such behavioral changes between being accommodating and threatening had also been noted by the staff during his stay at the ward. He had consumed alcohol and anxiolytics/sedatives at the time of the assault, and he stated that he was innocent. The records showed no contact with psychiatric care during his childhood, but as an adult, he had been treated for depression and anxiety. His parents divorced when he was 10, and he has had no contact with his father since. He has passing grades from primary school to high school and stated that he had friends and also had a few (although short) romantic relationships. He admitted substance use but denied drug problems. He was initially positive toward intellectual testing, but soon after starting such, he discontinued the testing due to it being a “bad test of intelligence” and a “waste of time.” The clinical impression during testing and the tests that were completed before his discontinuation indicated a normal (at least not significantly low) IQ level. He said that he often felt misunderstood and often wondered if people were out to get him or wanted to sabotage him. He also mentioned in the interviews that he used “markers” at his door to be sure that no one had entered the apartment when was not home or was sleeping and that he had a bulletproof vest at home, although it also became clear that he did not have a criminal lifestyle. This case vignette was meant to reflect the ideal type of a personality syndrome (including antisocial traits) by highlighting his grandiose, manipulating behavior and need for control. However, the symptoms of paranoia (depending on their severity and impact on reality monitoring where information was lacking) may nevertheless indicate an SMD.

#### Vignette 3

A 25-year-old man was charged with aggravated assault on his mother. There was no indication that he was under the influence of alcohol or drugs at the time, he denied current alcohol and drug use, and he did not talk about his feelings or thoughts concerning the assault. His life situation at the time of the assault was fraught with irritation. He was a probationary employee, and he thought that his boss was an “idiot” and that his workplace was too noisy. During the investigation, he largely only answered questions which needed a “yes”/“no” response. On the rare occasions that he made eye contact, the quality was perceived as peculiar (e.g., too intense or too erratic). His facial expressions were rather sparse, and he did not express either strongly negative or positive emotions. According to records, he lived with his mother, had never met his father, and had no current nor previous psychiatric contact. He had completed primary school with passing grades but discontinued high school since he did not like it there (he said the teachers and classmates were “stupid”). He had no current or historic romantic relationship and did not want to answer questions regarding friends. The general intellectual testing indicated a verbal intelligence level, the task processing speed was below the normal range, and there was a perceptual intelligence level within the normal range. The ideal type behind this case reflects a person who primarily has neuropsychiatric problems (e.g., autism), highlighting factors such as his impaired psychosocial functioning and normatively deviant social interaction patterns. However, the severity of the psychiatric symptoms, disturbed reality monitoring, and impaired psychosocial functioning (i.e., unclear factors in the vignette) could make an SMD possible in this case.

After reading each vignette, the participants were asked to list down the types of information that they would require to be able to make an SMD decision. They were also asked to note their preliminary assessment of SMD/no SMD and their preliminary psychiatric diagnostic hypothesis and present arguments for and against their SMD assessment and diagnostic evaluations.

In part 2 of the response form, based on a list with 18 different information sources (see Table 1), the participants marked all types that they often used while working with FPIs (including determining a diagnosis). All information sources were specified, apart from no. 18 (“other sources”), which gave the participants a chance to name themselves the information sources that they used and that had not been mentioned previously in the list.

**TABLE 1:** The sample’s and the professional groups’ use of information in general FPI-praxis (part 2), in the three cases (part 1), and in their perceived usefulness of the information sources (part 3).

	Type of information	Part 2: percent of participants who	Part 1: percent of participants who			Part 1, continued χ^2^ test; differences between	Part 3: the overall most
	source	used the information in general	requested the respective sources of			professions in requested information sources for	commonly reported
		FPI-praxis	information in each case			each case	alternative (%/*n*)
			
			Case 1	Case 2	Case 3		
1	Information (verbal and non-verbal) from interviews conducted by forensic social worker	85% (*n* = 35)	76 (*n* = 31 Pg: 10 Sw: 9 Pt: 12)	83 (*n* = 34 Pg: 11 Sw: 10 Pt: 13)	85 (*n* = 35 Pg: 11 Sw: 10 Pt: 14)	1: χ^2^ (2, *n* = 41) = 1.04, *p* = 0.595 2: χ^2^ (2, *n* = 41) = 1.61, *p* = 0.445 3: χ^2^ (2, *n* = 41) = 2.77, *p* = 0.250	6 (32%, *n* = 13)
2	Information (verbal and non-verbal) from an interview with a forensic psychologist	93% (*n* = 38)	95 (*n* = 39 Pg: 15 Sw: 9 Pt: 15)	95 (*n* = 39 Pg: 15 Sw: 9 Pt: 15)	95 (*n* = 39 Pg: 15 Sw: 9 Pt: 15)	1: χ^2^ (2, *n* = 41) = 5.73, *p* = 0.057 2: χ^2^ (2, *n* = 41) = 5.73, *p* = 0.057 3: χ^2^ (2, *n* = 41) = 5.73, *p* = 0.057	6 (41%, *n* = 17)
3	Information (verbal and non-verbal) from a medical interview (psychiatrist)	93% (*n* = 38)	95 (*n* = 39 Pg: 15 Sw: 9 Pt: 15)	93 (*n* = 38 Pg: 14 Sw: 9 Pt: 15)	90 (*n* = 37 Pg: 13 Sw: 9 Pt: 15)	1: χ^2^ (2, *n* = 41) = 5.73, *p* = 0.057 2: χ^2^ (2, *n* = 41) = 3.10, *p* = 0.211 3: χ^2^ (2, *n* = 41) = 2.72, *p* = 0.256	6 (56%, *n* = 23)
4	Observations from the ward where the person stayed during the investigation	93% (*n* = 38)	100 (*n* = 41 Pg: 15 Sw: 11 Pt: 15)	93 (*n* = 38 Pg: 14 Sw: 10 Pt: 14)	100 (*n* = 41 Pg: 15 Sw: 11 Pt: 15)	1: No analysis possible, all answered yes. 2: χ^2^ (2, *n* = 41) = 0.07, *p* = 0.966 3: No analysis possible, all answered yes	6 (44%, *n* = 18)
5	Results on subtests or full-scale values in intelligence tests	93% (*n* = 38)	39 (*n* = 16 Pg: 8 Sw:2 Pt: 6)	34 (*n* = 14 Pg: 9 Sw: 2 Pt: 3)	56 (*n* = 23 Pg: 11 Sw: 3 Pt: 9)	1: χ^2^ (2, *n* = 41) = 3.30, *p* = 0.192 2: χ^2^ (2, *n* = 41) = 7.04, *p* = 0.030*^a^* 3: χ^2^ (2, *n* = 41) = 5.61, *p* = 0.060	4 (34%, *n* = 14)
6	Results from psychological descriptive tests of cognitive functions	80% (*n* = 33)	49 (*n* = 29 Pg: 10 Sw: 4 Pt: 6)	44 (*n* = 18 Pg: 9 Sw: 4 Pt: 5)	68 (*n* = 28 Pg: 12 Sw: 6 Pt: 10)	1: χ^2^ (2, *n* = 41) = 3.06, *p* = 0.216 2: χ^2^ (2, *n* = 41) = 2.51, *p* = 0.285 3: χ^2^ (2, *n* = 41) = 1.92, *p* = 0.381	3 (37%, *n* = 15)
7	Results from psychiatric self-assessment forms	68% (*n* = 28)	54 (*n* = 22 Pg: 12 Sw: 2 Pt: 8)	59 (*n* = 24 Pg: 12 Sw: 4 Pt: 8)	51 (*n* = 21 Pg: 12 Sw: 2 Pt: 7)	1: χ^2^ (2, *n* = 41) = 9.75, *p* = 0.008*^a^* 2: χ^2^ (2, *n* = 41) = 5.24, *p* = 0.073 3: χ^2^ (2, *n* = 41) = 9.90, *p* = 0.007*^a^*	3 (32%, *n* = 13)
8	Results from performance-based tests that examine how the person processes stimuli and solves tasks	61% (*n* = 25)	54 (*n* = 22 Pg: 7 Sw: 5 Pt: 10)	24 (*n* = 10 Pg: 4 Sw: 1 Pt: 5)	61 (*n* = 25 Pg: 9 Sw: 6 Pt: 10)	1: χ^2^ (2, *n* = 41) = 1.61, *p* = 0.446 2: χ^2^ (2, *n* = 41) = 2.08, *p* = 0.352 3: χ^2^ (2, *n* = 41) = 0.40, *p* = 0.818	3 (37%, *n* = 15)
9	Results from projective tests that require association	29% (*n* = 12)	22 (*n* = 9 Pg: 4 Sw: 1 Pt: 4)	15 (*n* = 6 Pg: 3 Sw: 1 Pt: 2)	32 (*n* = 13 Pg: 3 Sw: 4 Pt: 6)	1: χ^2^ (2, *n* = 41) = 1.45, *p* = 0.484 2: χ^2^ (2, *n* = 41) = 0.63, *p* = 0.727 3: χ^2^ (2, *n* = 41) = 1.53, *p* = 0.464	7 (27%, *n* = 11)
10	Reports from the police (for example the person’s behavior at the crime scene, at the time of arrest, in custody)	90% (*n* = 37)	98 (*n* = 40 Pg: 14 Sw: 11 Pt: 15)	98 (*n* = 40 Pg: 14 Sw: 11 Pt: 15)	90 (*n* = 37 Pg: 11 Sw: 11 Pt: 15)	1: χ^2^ (2, *n* = 41) = 1.77, *p* = 0.411 2: χ^2^ (2, *n* = 41) = 1.77, *p* = 0.411 3: χ^2^ (2, *n* = 41) = 7.68, *p* = 0.021*^a^*	6 (44%, *n* = 18)
11	Reports from prosecutors	34% (*n* = 14)	27 (*n* = 11 Pg: 4 Sw: 4 Pt: 3)	27 (*n* = 11 Pg: 4 Sw: 4 Pt: 3)	22 8 (*n* = 9 Pg: 3 Sw: 3 Pt: 3)	1: χ^2^ (2, *n* = 41) = 0.86, *p* = 0.649 2: χ^2^ (2, *n* = 41) = 0.86, *p* = 0.649 3: χ^2^ (2, *n* = 41) = 2.48, *p* = 0.883	8 (24%, *n* = 10)
12	Reports from lawyers	22% (*n* = 9)	17 (*n* = 7 Pg: 4 Sw: 1 Pt: 2)	17 (*n* = 7 Pg: 4 Sw: 1 Pt: 2)	17 (*n* = 7 Pg: 3 Sw: 1 Pt: 3)	1: χ^2^ (2, *n* = 41) = 1.61, *p* = 0.445 2: χ^2^ (2, *n* = 41) = 1.61, *p* = 0.445 3: χ^2^ (2, *n* = 41) = 0.67, *p* = 0.713	7 (32%, *n* = 13)
13	Reports from witnesses or other third parties related to the crime	88% (*n* = 36)	98 (*n* = 40 Pg: 14 Sw: 11 Pt: 15)	93 (*n* = 38 Pg: 15 Sw: 10 Pt: 13)	83 (*n* = 34 Pg: 15 Sw: 6 Pt: 13)	1: χ^2^ (2, *n* = 41) = 1.77, *p* = 0.411 2: χ^2^ (2, *n* = 41) = 2.03, *p* = 0.361 3: χ^2^ (2, *n* = 41) = 9.49, *p* = 0.009*^a^*	5 (44%, *n* = 18)
14	Reports from interviews with relatives or other third parties related to the person’s functional level	85% (*n* = 35)	90 (*n* = 37 Pg: 15 Sw: 9 Pt: 13)	78 (*n* = 32 Pg: 13 Sw: 11 Pt: 8)	98 (*n* = 40 Pg: 15 Sw: 10 Pt: 15)	1: χ^2^ (2, *n* = 41) = 2.72, *p* = 0.256 2: χ^2^ (2, *n* = 41) = 9.09, *p* = 0.011*^a^* 3: χ^2^ (2, *n* = 41) = 2.79, *p* = 0.247	4 (39%, *n* = 16)
15	Reports from interviews with relatives or other third parties related to the person’s personality	71% (*n* = 29)	68 (*n* = 28 Pg: 12 Sw: 6 Pt: 10)	76 (*n* = 31 Pg: 13 Sw: 7 Pt: 11)	78 (*n* = 32 Pg: 12 Sw: 9 Pt: 11)	1: χ^2^ (2, *n* = 41) = 1.92, *p* = 0.381 2: χ^2^ (2, *n* = 41) = 1.89, *p* = 0.388 3: χ^2^ (2, *n* = 41) = 0.31, *p* = 0.852	3 (37%, *n* = 15)
16	Physical examination	63% (*n* = 26)	24 (*n* = 10 Pg: 4 Sw: 1 Pt: 5)	20 (*n* = 18 Pg: 2 Sw: 0 Pt: 6)	27 (*n* = 11 Pg: 3 Sw: 0 Pt: 8)	1: χ^2^ (2, *n* = 41) = 2.08, *p* = 0.352 2: χ^2^ (2, *n* = 41) = 7.04, *p* = 0.030*^a^* 3: χ^2^ (2, *n* = 41) = 9.75, *p* = 0.008*^a^*	2 (39%, *n* = 16)
17	Biological factors (for example, drug trials, EEC, brain imaging studies)	83% (*n* = 34)	49 (*n* = 20 Pg: 10 Sw: 6 Pt: 9)	63 (*n* = 26 Pg: 9 Sw: 8 Pt: 9)	46 (*n* = 19 Pg: 8 Sw: 4 Pt: 7)	1: χ^2^ (2, *n* = 41) = 0.40, *p* = 0.818 2: χ^2^ (2, *n* = 41) = 5.62, *p* = 0.755 3: χ^2^ (2, *n* = 41) = 0.736, *p* = 0.692	3 (37%, *n* = 15)
18	Other factors (specify)					Not included	

*Red (2), rarely useful; orange (3), sometimes useful; yellow (4), often useful; light green (5), almost always useful; dark green (6), always useful; light blue (7), source not used; blue (8), do not know if this source is useful or not; Pg, forensic psychologist; Sw, forensic social worker; Pt, forensic psychiatrist.*

*^a^Alpha-level set to p < 0.05, but after Bonferroni correction for multiple comparisons, p = 0.003. No values reached a statistical difference after Bonferroni correction.*

In part 3, the participants ranked the information sources in the 18-item list mentioned above according to how much they considered each source to have been of help in their FPIs during the past year. Each source was rated on a scale between 1 and 6 (anchors: 1 = never useful, 6 = always useful) or answered by ticking either of two boxes: “source not used” or “don’t know if this source is useful or not”. In part 3, the participants specified, for each source and in short formulations, how this information source had been useful in FPIs (e.g., “having had previous treatment contact with psychologist” or “mental illness in the family”).

The list of information sources was based on information sources commonly used in FPI praxis (e.g., psychiatric journals, documents from the criminal investigation), but the formulation of the sources was then guided by a previous research on state-of-the-art psychological assessment (19) to include life data (information about the person’s life, such as marriage and children, life events, and education), self-report data (information that the individual shares about himself—for instance, *via* psychiatric journal and in interviews), test data (information from completed tests), and observation data (observations of the person, such as referent interviews with physicians, teachers, or relatives).

### Procedure

The data was collected principally for 2 weeks: the first week on two consecutive days at one of the departments of DPF and the subsequent week on two consecutive days at the other department. The participants were instructed to not discuss the interview questions and vignettes with co-workers until the entire data collection was completed to minimize external influence on the answers. To further minimize this risk, the data collection was carried out during as few days, as closely together, as possible. Since some experts expressed interest but were unavailable on the specific dates, eight participants were included after these two data collection weeks. Adding eight more participants was considered important enough to risk minor contamination of them as data sources since statistical power was critical.

Before the participants read the vignettes and answered its response form, they also participated in a semi-structured interview [see Svensson et al. (40) for more information]. Before the interview, the participants were given information about the purpose of the study and signed the informed consent form. After the interview, the participants received the vignettes and response form (answered alone in a secluded room). The concluding participation took approximately 1.5 h, after which the participants were instructed to put their individually coded response forms in a blank envelope and put this in a sealed letter box which was emptied after their participation (all participants gave answers to this form).

### Data Analysis

Statistical analyses were conducted (SPSS 26) on the use of information sources to identify which sources the FPI experts (1) required for each of the three case vignettes and (2) had used in their FPI praxis during the past year and (3) the perceived usefulness of each information source during that year. The alpha level was set to *p* < 0.05 using Bonferroni correction when required. The grouping by professional experience was restructured to obtain numerically more equal group sizes. For part 1, a between–within-subjects ANOVA was used to examine the same information sources’ perceived relevance to the three cases. The dependent variable was the number of information sources requested for each case. To analyze the consensus between professions and cases regarding which information source to base their decisions on in the respective cases, Cohen’s Kappa was also used. The Kappa values were interpreted based on McHughs (41) approach: 0–0.20 = no/negligible agreement, 0.21–0.39 = minimal agreement, 0.40–0.59 = weak agreement, 0.60–0.79 = moderate agreement, 0.80–0.90 = strong agreement, and ≥90 = almost perfect agreement. A χ^2^ test for independence was also performed for each vignette case to explore potential differences in the proportions of the professional group’s opinion regarding suspected SMD/no SMD. For part 2, a one-way ANOVA was used to investigate the number of information sources that the professions reported to have used during the previous year. Three participants had missing values and were excluded from this analysis. For part 3, Kruskal–Wallis *H*-test was performed regarding how useful the different professions perceived the various information sources used during the previous year to be.

## Results

**FIGURE 2:**
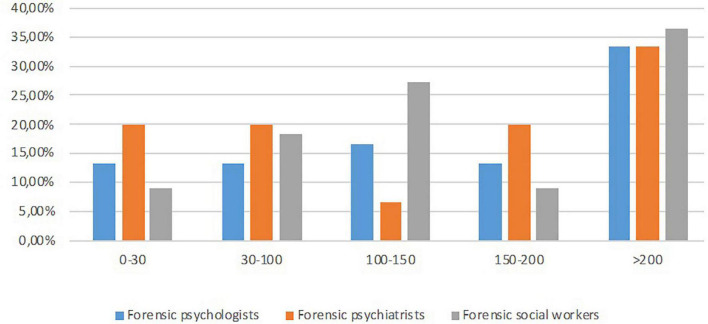
Percent of participants by profession and experience.

In terms of profession and level of experience (see Figure 2), two χ^2^ goodness-of-fit tests indicated no significant differences in the proportion of profession groups by experience level represented in the sample, χ^2^ (8, *n* = 41) = 3.32, *p* = 0.913, and no significant difference regarding the represented professions’ level of experience, χ^2^ (4, *n* = 41) = 5.46, *p* = 0.243. However, the tests of normality showed that the variable experience was not normally distributed (all Shapiro–Wilk > 0.775, all *p* < 0.062). This variable was therefore not used as an independent variable in the analyses.

### Part 1: Differences Between the Professions’ Use of Information in the Three Cases

**FIGURE 3:**
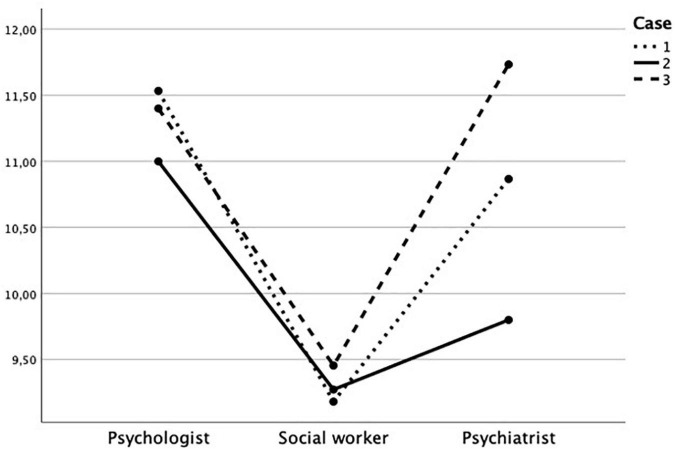
Mean values of information sources requested by each profession in each case.

Part 1 incorporated research questions 1–3. Numerically, forensic psychologists requested most sources of information in all three case vignettes, while forensic social workers requested the least, and psychiatrists varied the most between the case context in how many information sources they requested (see Table 2 and Figure 3). The tests of normality for the number of information sources requested in cases 1, 2, and 3, respectively (i.e., three variables), were not significant (all Shapiro–Wilk > 0.924; all *p* > 0.223).

**TABLE 2:** Mean values (and standard deviations) of number of information sources in each case vignette by profession and experience.

Case	Profession			Experience				
		
	Forensic psychologist (*n* = 15)	Forensic social worker (*n* = 11)	Forensic psychiatrist (*n* = 15)	1–30 (*n* = 6)	30–100 (*n* = 7)	100–150 (*n* = 8)	150–200 (*n* = 6)	>200 (*n* = 14)
Case 1	11.53 (3.2)	9.18 (3.45)	10.86 (2.97)	11.50 (3.56)	10.00 (2.08)	9.12 (2.74)	9.83 (4.07)	11.85 (3.37)
Case 2	11.00 (3.09)	9.27 (2.83)	9.80 (2.98)	10.00 (2.96)	10.14 (1.95)	8.37 (3.24)	10.16 (3.92)	11.07 (2.84)
Case 3	11.40 (3.11)	9.45 (2.42)	11.73 (2.63)	11.16 (3.12)	11.14 (3.18)	9.62 (2.61)	11.16 (3.06)	11.57 (2.82)

**FIGURE 4:**
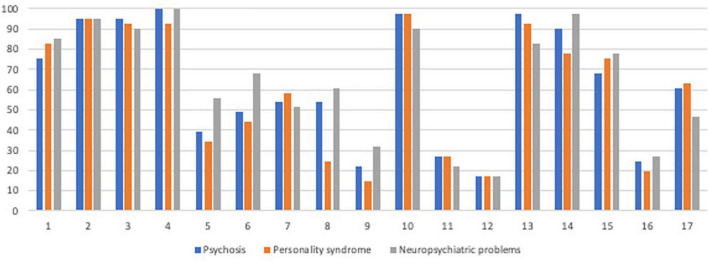
Percentage of participants who requested the information source per case.

A between–within-subjects ANOVA was conducted to investigate if the professions differed regarding how many information sources they requested in each of the three cases (between-subjects factor: profession; within-subjects factor: number of requested information sources for cases 1, 2, and 3, respectively) (see Figure 4). The results of Box’s test and Levene’s test were not significant for either case (all *p* > 0.52). The multivariate test showed a significant main effect and a large effect size for type of case [Wilks’ Lambda = 0.76, *F*(2,37) = 5.65, *p* = 0.007, η^2^ = 0.23]. There was no significant interaction between type of case and profession [Wilks’ Lambda = 0.78, *F*(4,74) = 2.42, *p* = 0.056, η^2^ = 0.12]. The result of the univariate test of within-subjects effects for type of case was significant [*F*(2,76) = 3.46, *p* = 0.037, η^2^ = 0.08]. The result of the univariate test of between-subjects effects for profession was not significant [*F*(2,38) = 1.77, *p* = 0.184, η^2^ = 0.09]. Taken together, the professional groups wanted to use the most information sources in case 3 and the least in case 2, but the professional groups did not differ significantly regarding how many they requested.

Different types of information were relevant for different cases. The Kappa statistic was used to determine an expert’s consistency over the three cases regarding which types of information were considered relevant. Due to the many comparisons made, differences found at *p* = 0.05 between cases regarding information sources were reported here for transparency’s sake, but only those which met the Bonferroni-corrected alpha-level were referred to as significant (adjusted: *p* = 0.003; 17 analyses made—see Figure 4 for an overview of all results). The significant differences are outlined below.

#### Reports From Relatives/Third Party: Personality and Functioning

Regarding source 13, reports from witnesses/third party about the crime, a minimal agreement between cases 2 and 3 was found (ϰ = 0.33), with this source being considered the most important in case 1, but it was not significant after correction (*p* = 0.018). Regarding source 14, interview with relatives/other third party regarding functional level, a significantly minimal agreement between cases 1 and 3 was found (ϰ = 0.38, *p* = 0.002), where this source was often deemed relevant for case 3 but not for case 1. Regarding source 15, interview with relatives/other third-party regarding personality, this differed significantly between both cases 1 and 2 (ϰ = 0.70, *p* < 0.001; more relevant to case 2) and between cases 2 and 3 (ϰ = 0.66, *p* < 0.001; more relevant for case 3). Although the agreement was also minimal between cases 1 and 3 (more relevant for case 3), this was not significant after Bonferroni correction (ϰ = 0.39, *p* = 0.011).

#### Tests of Cognitive Functioning

Regarding the use of source 5, results from intelligence test, significantly weak agreements were found between cases 1 and 2 (ϰ = 0.48, *p* = 0.002) and between cases 2 and 3 (ϰ = 0.48, *p* = 0.001) but with only a non-significant minimal agreement between cases 1 and 3 (ϰ = 0.38, *p* = 0.009). Regarding question 6, descriptive psychological tests of cognitive functions, significantly weak agreements were found between all cases: 1 and 2 (ϰ = 0.51, *p* = 0.001), 1 and 3 (ϰ = 0.52, *p* < 0.001), and 2 and 3 (ϰ = 0.44, *p* = 0.001). These two sources were considered most important in case 3, less so in case 1, and the least in case 2. Regarding source 8, performance-based tests, a significantly weak agreement was found between cases 1 and 3 (ϰ = 0.46, *p* = 0.003). These low levels of agreement were due to the source being considered more important in case 3 than in case 1. Although the agreement was also minimal between cases 1 and 2 (ϰ = 0.34, *p* = 0.008; more relevant for case 1 than for case 2). This was not significant after Bonferroni-correction.

#### Self-Report Forms

Regarding source 7, results from psychiatric self-report forms, its importance differed between cases 1 and 2 (ϰ = 0.70, *p* < 0.001; significant and with varying agreement), between cases 1 and 3 (ϰ = 0.56, *p* < 0.001), and cases 2 and 3 (ϰ = 0.46, *p* = 0.003; both significant and with weak agreement). Taken together, this source was considered most important in case 2, less so in case 1, and the least in case 3.

#### Reports From Police

Regarding source 10, reports from police, this information was considered less important in case 3 compared to those in cases 1 and 2 (both significant comparisons: ϰ = 0.38, *p* = 0.002). However, a significant and perfect agreement was found between cases 1 and 2 (ϰ = 1.0, *p* < 0.001; all participants considered this information source important in these two cases).

To ascertain whether there were differences among the three professional groups regarding which information sources they requested in each of the three cases, χ^2^ tests were performed. Significant results were found for six information sources—for example, psychologists and psychiatrists requested intelligence testing results more often in case 2 than the other professions did (*p* = 0.030), psychologists and psychiatrist requested self-reported psychiatric symptom forms in case 1 (*p* = 0.008) and case 3 (*p* = 0.007) more often than social workers did, and psychiatrists requested physical examination more often in case 2 (*p* = 0.030) and case 3 (*p* = 0.008) than the other professions did (see Table 1 for all results). However, none was significant after Bonferroni correction (*p* = 0.003).

#### Different Professions’ Conclusion Regarding SMD

To examine the professions’ preliminary assessment regarding SMD at (a) the time of the crime and (b) the time of the FPI, a χ^2^ test for independence was performed for each case. Only in case 1 (i.e., ambiguous psychosis) was a difference (at *p* = 0.05) found between the professions regarding SMD at the time of the FPI: forensic psychologists leaned toward SMD more often than expected [χ^2^(1,41) = 4.90, *p* = 0.030] and forensic social workers leaned toward SMD less often than expected [χ^2^(1,41) = 5.88, *p* = 0.020] (see Table 3 for distributions). However, these differences were not significant after Bonferroni correction (adjusted to *p* = 0.008; six analyses were made). Furthermore, regarding case 1, forensic psychologists and psychiatrists were more consistent in their leaning towards SMD both at the time of the crime and the FPI, while forensic social workers were more divided between for/against SMD. A similar pattern was found in case 3 (i.e., ambiguous neuropsychiatry), but in case 2 (i.e., ambiguous personality disorder), all professions were consistent in their leaning predominantly against SMD (see Table 3).

**TABLE 3:** Percentage of the professional groups’ assessment regarding severe mental disorder in each case at the time of the crime and at the forensic psychiatric investigations.

	Case 1		Case 2		Case 3	
			
	The crime (yes/no)	The FPI (yes/no)	The crime (yes/no)	The FPI (yes/no)	The crime (yes/no)	The FPI (yes/no)
Forensic psychologist	93%/7%	93%/7%	7%/96%	0%/100%	67%/33%	60%/40%
Forensic psychiatrist	73%/27%	73%/27%	7%/96%	7%/93%	60%/40%	53%/47%
Forensic social worker	64%/36%	45%/55%	18%/82%	18%/82%	45%/55%	45%/55%

### Part 2: Information Sources Used in FPIs During the Previous Year

Part 2 incorporated research question no 4. A one-way ANOVA was conducted to investigate the number of information sources that the different professions reported to have used in their investigations during the previous year (forensic psychologist: *M* = 13.10, SD = 2.56; forensic psychiatrist: *M* = 13.53, SD = 2.03; forensic social worker: *M* = 13.50, SD = 3.10). The main effect of profession was not significant [*F*(2,35) = 0.13, *p* = 0.877, η^2^ = 0.01].

### Part Three: The General Usefulness of Various Information Sources

**FIGURE 5:**
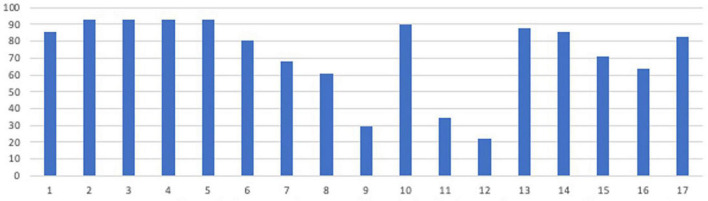
Percentage of participants who considered the information source important in FPIs in general.

Part 3 incorporated research question number 4 (see Table 1 and Figure 5 for an overview). Kruskal–Wallis *H*-test was used to identify significant differences between professions regarding the perceived usefulness of different information sources in their general FPI praxis. Due to the many comparisons, only questions with significant differences between professions were reported here (all others, non-significant, Kruskal–Wallis test: *H* < 3.48, *p* > 0.175). The significant differences between professions found with Kruskal–Wallis test were explored with *post hoc* comparisons using Dunn’s pairwise test and a Bonferroni-adjusted *p*-value (see Table 1). There were differences among professions regarding source 1 [interview with forensic social worker, χ^2^ (2,38) = 9.00, *p* = 0.011], and Dunn’s pairwise test showed that forensic social workers perceived this source as more useful than forensic psychologists did (*p* = 0.008). Significant differences were also found regarding source 2 [interview with forensic psychologist, χ^2^ (2,38) = 9.83, *p* = 0.007], where forensic psychologists perceived this information as more useful than forensic psychiatrists did (*p* = 0.008). Regarding source 9 [use of projective tests, χ^2^ (2,19) = 7.40, *p* = 0.025], it was shown that although all professions perceived this source as generally not useful in FPIs, forensic psychologists perceived this source as even less useful than forensic social workers did (*p* = 0.020).

Regarding source 12 [reports from lawyer, χ^2^ (2,18) = 7.40, *p* = 0.025], forensic psychologists perceived this source as more useful than forensic psychiatrists did (*p* = 0.028). Regarding source 17 [biological factors, χ^2^ (2,36) = 10.88, *p* = 0.004], Dunn’s pairwise test showed that forensic psychologists considered this information as more important than both forensic psychiatrists (*p* = 0.048) and forensic social workers (*p* = 0.007) and also that forensic psychiatrists generally perceived this source as not useful, while forensic psychologists and forensic social workers were more positive.

## Discussion

Regarding research questions 1–3, the present study showed that the FPI experts adapted their use of various types of information sources to the different types of cases, but on an overarching level, no significant result regarding the number of information sources used in the case vignettes by different professional groups was found. There were indications that professional groups (here psychologists and social workers) differed in whether they leaned toward SMD or not in case 1 (hereafter ambiguous psychosis). Numerically, most participants suspected SMD in the ambiguous psychosis case, and least suspected SMD in case 2 (hereafter ambiguous personality disorder). However, in case 3 (hereafter ambiguous neuropsychiatry), the experts from all professional groups were more equally divided for/against SMD (approximately 50–50%/60–40% division between for/against suspected SMD) at this stage of the case vignette. Regarding research question 4, regarding the use and perceived usefulness of information sources in FPIs during the previous year, a profile was found with minor numerical differences, which means that the professional groups overall agreed regarding the use and how useful they thought the different information sources were, again with some minor differences. Since some information sources were only considered to be useful sometimes in general FPI praxis, the relevance of case context and case-specific adaptation of the FPI was highlighted by these results.

### Impact of Case Context on the Use of Information Sources in FPIs

In all three cases, observations from the FPI ward were almost unanimously requested by all participants. Hence, regardless of the type of case and profession, how the person behaved at their time at the ward was considered important information. This is hardly surprising since observations from the ward are relevant to all three professional groups’ assessment (e.g., staff–client interactions, verifying self-reported information such as psychiatric symptoms through actual behavior). This result contributes to the international research field on forensic psychiatric decision-making, but international comparisons are needed to investigate the importance of this information source in other countries with different kinds of FPI praxis, especially where FPI experts assess the client at a jail or in an ordinary psychiatric ward. This also actualizes the question regarding FPIs that are conducted when the client is not in custody, a praxis that differs within countries. If information from the FPI ward is considered to be such a central information source, it would be important for future research to investigate how experts conducting FPIs with clients who are not in custody compensate for this lack of clinical observation information.

Other information sources were considered more relevant in certain case contexts than others, such as interview with relatives/other third-party regarding personality, which was deemed less relevant in the ambiguous psychosis case than in the other two cases, and also various psychological tests of cognitive functioning which were deemed more important in the ambiguous neuropsychiatry case than in the others. Conversely, information from psychiatric self-report forms was considered most important in the ambiguous personality disorder case compared to that in the ambiguous neuropsychiatry case. Considering the nature of the information source, this indicates that it was more important to ascertain the severity of psychiatric symptoms (e.g., whether the paranoid symptoms should be considered on a psychotic level or not) in the ambiguous personality disorder case than in the ambiguous neuropsychiatry case. It could also indicate that the information regarding psychiatric symptoms obtained specifically by self-report would be less informative for the ambiguous neuropsychiatry case than for the other cases. Information in reports from police were also considered more important (by all professions) in the ambiguous psychosis case and personality disorder case, indicating that observations of behavior during the arrest and/or transcripts of interrogations were especially important in these types of cases compared to when neuropsychiatric problems are suspected. Taken together, these results could be interpreted in light of differences in *why* the different case characteristics could be considered as SMD. For psychosis or a paranoid reaction of psychotic magnitude, it would be central to have information on how the person behaved at the crime scene (e.g., were delusional ideas expressed, was the person disoriented, etc.), which is often included in police reports. For neuropsychiatric and/or suspected intellectual disability, the SMD decision is more related to the severity of impaired functioning in several areas (e.g., cognitive capacities, everyday functioning), which are ascertained more accurate by testing during the FPI or by interviewing referents (e.g., parents, staff at the client’s housing facility).

#### Differences Between Professions on the Use of Information Sources

Regardless of the type of case, forensic psychologists generally requested the most information sources, and forensic social workers generally requested the least. Among the forensic psychiatrists, there was more case-related variation (i.e., most for the ambiguous neuropsychiatry case and least in the ambiguous personality disorder case). Otherwise, information source-specific discrepancies between professions were indicated—for example, that social workers did not request intelligence tests to the same extent as psychologist and psychiatrists. The reason for these exploratory patterns in the present study could be due to the fact that forensic psychiatrists and psychologists should have more diagnostic focus in their assessments (i.e., assessments should include cognitive functioning, medical history) (33, 34). Furthermore, forensic social workers, in general, should focus on the client’s psychosocial functioning, which manifests itself most clearly through their present and historical social interventions, thus comprehensively described in documents from the social services. Social services documentation is multifaceted and contains a wealth of information about various areas of functioning (e.g., economy, having been in social services’ custody as a child, whether their parents had required welfare support during their childhood) and also on stays in treatment facilities for substance abuse paid for by social services (i.e., documentation often includes a care journal from such treatment facilities) (32). Hence, even though forensic social workers requested less information sources, the difference was minor, and the source that they primarily request may in itself include a number of life history information sources. If the professional group’s information seeking routines have been developed or routinely adapted over time to the guiding documents (32–35) and the clinician does not consider the information selection carefully in each case, this could lead to bias in requesting what is “always done” and failure to evaluate the need for the information source based on specific case characteristics [see HEP hierarchy; (16)]. Due to the observed variations over the different cases for certain types of information (e.g., intellectual testing), the risk of this kind of bias seemed relatively low in the present study, but if a routine has emerged, extraneous information could be collected, which could create bias due to decision-making being affected by case-irrelevant information. Since it is not always known exactly which kind of information will be available in various registries, the experts need to balance the risk of collecting extraneous information against missing the inclusion of possibly important information. Since this may vary between cases, no general guideline can be established. However, for an expert to always know why he/she is requesting a certain type of information in *the specific case* would be the bare minimum criterion to mitigate such a risk.

#### How to Proceed With Further Investigation

The participants were only given limited information in the cases, and they requested information from several different sources, including a self-report perspective (i.e., the client’s), observation perspective (e.g., from the ward, from referents), test perspective (i.e., standardized test results), and a life perspective (e.g., records from criminal, medical, social registries) [(19); see also (42)]. To use this type of strategy, collecting data from multiple perspectives using their respective methods has been presented as best practice to get a more nuanced clinical picture and as a way of diminishing the impact of bias (19, 20). Information from different sources, methodologically and theoretically, increases the chance of contradictions within the data, which increases the chance of type 2 processing and thereby the possibility to make well-founded decisions. Various information sources from different perspectives were requested by experts in the present study, indicating a diminishing of risk-making decisions based on insufficient data and type 1 processes (8). This praxis should be considered an important aspect of evidence-based decision-making within FPIs. However, to investigate whether there is no structural bias regarding what kind of weight these information sources are assigned, qualitative studies on these processes are needed.

#### Differences in the Inclinations of Professional Groups Regarding SMD

Regarding the ambiguous psychosis case, forensic psychologists leaned toward SMD at the time of the FPI more often than expected, while forensic social workers did so less often than expected. Even though this difference was not significant after Bonferroni correction, it was considered important to note for future research, especially since there were indications of more similarity in SMD leanings between forensic psychologists and psychiatrists than among forensic social workers who were more divided between for/against SMD. This difference between professional groups concerned primarily the ambiguous psychosis case and ambiguous neuropsychiatry case since all professional groups were consistent in their leaning predominantly against SMD in the ambiguous personality disorder case. Based on earlier findings (17) and Dror (2) categorization of types of sources of bias, the possible reason for such differences between professions could be explained by the bias occurring due to education and training, the professionals interpreting the case using different perspectives based on their professional training (2, 17), or due to praxis developed to suit the DFP assessment guidelines—for example, the forensic social workers may, in general, not have considered the level of psychosocial functioning as sufficiently impaired in the ambiguous psychosis case, while the other two professions who bases their SMD decision on the assessment of more similar factors (e.g., psychiatric symptoms, cognitive profile, personality functioning) were more in agreement regarding SMD. This highlights both the positive and negative aspects of the FPI team structure. As mentioned, the Swedish teamwork with three to four professional groups routinely participating in the FPI assessment praxis is likely unique (28). Since the results indicated that the professional groups sometimes differ regarding their view on SMD, this could indicate that each profession indeed investigates and analyzes SMD from different perspectives (biological, psychological, and social). If so, each profession could contribute with a different knowledge from the perspectives of the biopsychosocial model on mental health [e.g., (43)] within the decision-making process regarding SMD, which, in turn, may increase the chance of a more holistic assessment in the final report written by the forensic psychiatrist. Nevertheless, since SMD is a dichotomous concept in Sweden, such differences between professionals could create problems in the team’s general decision-making process if the different perspectives are not clearly described in such discussions and in reports. Since the lack of consistency in conclusions between FPI experts is considered to be generally negative (1, 42), different SMD conclusions from different professional groups could also be complicated for the court when a decision must be taken in a specific case.

### General Use of Information Sources and Their Perceived Importance in FPIs

Changing focus from the case vignette results into the general use of information sources in FPI praxis, the professional groups did not differ regarding how many information sources they had used in FPIs during the past year. The fact that the groups generally used similar types of information sources as in the case vignettes could be considered positive. Information considered important/valid to the professional groups’ decisions within FPIs generally overlap those used when FPIs are framed in various contexts, and if professionals also do not base their decisions in widely varying sources, it should increase the chance of the team being receptive to other professions’ conclusions. Interestingly, comparing part 1 and part 2 results, a certain discrepancy occurred between what information sources the different professions requested in specific cases and which they used in general. The information sources less often requested in the three case vignettes than in general FPI praxis concerned, first, various cognitive test results and, second, physical examination. There could be several reasons for this difference. First, experts could believe that they request this information, but actually do not, or that all case vignettes were perceived as being in a relatively “initial stage” of the FPI, and these three sources could be considered more relevant at a later stage of the FPI (e.g., for more nuanced differential diagnosis). What speaks for the latter explanation is that, when considering the results from part 3, these information sources were, on average, perceived by participants to be “useful sometimes” (i.e., only in some cases). In other words, based on part 2 and part 3, some sources may not be considered important in all FPIs, but to test certain differential diagnostic decisions relevant for SMD hypotheses, they could be of central importance. Based on these results, there seems to be a group of “core” information sources which are almost always considered important in all types of cases (e.g., to talk to the client), while others (e.g., tests of cognitive functioning) are important only in certain types of cases. Therefore, based on this information, it would not be advisable to create standardized FPI guidelines on the level of specific interventions (e.g., always interview a certain category of referents, always conduct a certain kind of psychological testing, etc.) since this could increase the risk of routinely collecting extraneous information that would not contribute relevant insights to the case but only increase the risk for bias.

Overall, experts collect and use a large amount of information sources in FPIs, and when processing this large amount of multifaced information, the risk for bias introduced by irrelevant case information increases, and they are also likely to experience a high cognitive load which pose another risk for biased decision-making. When considering the case specific-level of the taxonomy (2), this may not be the biggest problem in Swedish FPIs since the FPI experts actually did differ in their assessments between cases through adaptation (i.e., did not use the same approach in all case types). However, a potential general bias regarding how experts proceed with investigating a specific type of case (i.e., according to the kind of psychiatric problem) could nevertheless be relevant (e.g., having one specific approach when suspecting psychosis and another for autism). This aspect should be investigated further in future studies. The risk for bias due to a high cognitive load seems greater in Swedish FPIs due to the time limit (maximum: 4 weeks) and to the complexity of the cases [i.e., high cognitive load both to organizational level and case-level factors within the decision-making process; see HEP model (2)]. As has been observed in the results from the interviews preceding the vignette (40), the professionals considered stress to be one of the most detrimental aspects for their decision-making in FPIs, such as not having time to gather all information that one would, under less stressful circumstances, have done. Stress increases the risk of type 1 processes [including increasing the risk of bias (8)], and since there is a limited number of forensic experts working with FPIs (44) an increased workload could affect their decisions due to sometimes conducting more (and sometimes less) FPIs in parallel. Based on the current results, where many information sources were requested, it is likely that when experiencing a cognitive load or high stress levels, experts could be motivated to reach a conclusion fast, decreasing their motivation/ability to gather information from all these sources or listen to contradictory evidence (45). This could increase the risk for bias by limiting the amount of information to a restricted number of perspectives, and the conclusions risk being premature. Hence, even though the FPI experts’ inclination of gathering information from several different perspectives could reduce the risk of bias, such as tunnel vision [see (45)], a high cognitive load due to stress could decrease this ambition.

#### The Information Sources’ Perceived General Usefulness

In general, in FPI praxis, from a methodological assessment perspective (20), experts tended to consider information regarding self-report data and observation data as the most useful. Other useful information sources, but less consistently considered so, were intelligence tests (i.e., test data) and referent conversations with relatives/third party (i.e., observation data). The results from part 3 also indicated that the psychologists and social workers valued their own interviews more than the other profession’s to reach their conclusions. This could be considered natural since the objective of their respective reports is to base their decisions on their professional perspective. However, it could also be an indication that each profession tends to value their own contribution more than the contribution of others (e.g., blind spot bias, in-group preference). This can be linked to the findings of Neal and Brodsky (10) regarding the professionals making forensic psychiatric assessments perceiving themselves as less vulnerable to bias and therefore relying too much on their own work compared to that of others. Similar results were obtained in Commons et al. (15) where forensic psychiatrists markedly underestimated their own biases compared to their peers. Since Neal and Brodsky (10) did not include any other profession in their study, it is not possible to know whether this was an issue. To diminish the effect of the blind spot bias, the cross-professional team discussions (a core aspect of Swedish FPI praxis) of this entails a discussion of data collection and conclusions and comparing results and impressions. At least in theory, this should increase the opportunity for new interpretations of obtained results from other professional perspectives, in turn increasing the chance that one’s original hypothesis is questioned, activating type 2 processing (8). Hence, teamwork could be seen as an advantage in Sweden’s approach to FPI praxis since this could decrease the risk for certain bias, but if the team members rely most on their own opinion anyway and are not really open to changing their mind in light of new data from other professions, the beneficial effect of team discussions on increasing analytical type 2 processing (i.e., forcing the professional to try a change of perspective on the obtained assessment results, testing alternative explanations) would be lost. An open and non-judgmental atmosphere, something that decreases with stress, could therefore be considered a cornerstone for teamwork to increase the chance of evidence-based decision-making within FPIs. Although teamwork could be considered an advantage, it must be noted the experts regardless are exposed to other kinds of HEP model types of bias, due to human nature (e.g., fatigue, antipathy/sympathy toward a client), that could influence the forensic expert when conducting a FPI (1, 2), and also due to processes such as group think (e.g., inflated sense of certainty when ideas are endorsed by the group). These processes therefore need further investigation. More overarching organizational issues regarding FPIs and their relationship to increase or decrease the risk for bias also need further research—for example, a potential advantage in Sweden’s as well as Finland’s and Portugal’s [see (28)] approach to FPI praxis could be that forensic assessment experts are not retained by one party within a criminal case, as in the United States, but are employed by a governmental authority separate from the courts. Such an organizational structure could diminish pressure on the experts to reach specific conclusions in FPIs [e.g., lowering the risks for bias due to no relationship to parties who want the expert to “support their side”; see (4)]. When the expert is not paid per conducted FPI (i.e., the expert is employed by the government as an available resource to the courts), the risk of bias due to stress could potentially be also lowered.

### Limitations and Future Directions

By using the case vignette method, all participants were exposed to the same contexts and got to appraise the use of different information sources providing the conditions for examining reliability both within and between professional groups. However, a list of options regarding information sources was used instead of asking about free text responses, which could have affected—directed or impaired—the pattern of information sources reportedly used. To decrease this risk, a pilot study including representatives of each profession was conducted to capture factors missed by the researchers, and the participants were also given an opportunity to give a free text response if they used other information sources (i.e., “other factors”). Since only a few participants used this option, the risk of having missed important information sources used in FPIs is considered small. Although participation was anonymous, the number of participants was quite small, and it is possible that the participants may have adjusted answers based on social desirability (e.g., due to the risk of being recognized). Unfortunately, there was no way to investigate this factor in the present study, but due to the obtained variation within professional groups, this could be considered a less important risk.

## Conclusion

This study contributes empirical data to further the evidence-based decision-making praxis in FPIs. Although there was a core profile of the types of information sources usually requested in all three case vignettes, such as interviewing the client and observations from the ward, the FPI experts made some case-specific adaptations—for example, with psychosis and personality disorder, reports from the crime scene were considered especially important, while for neuropsychiatry, it was the level of cognitive and everyday functioning. This could also be related to the Swedish law regulating which psychological conditions can be considered as SMD and why. Differences in leanings toward/against SMD were found. The forensic social worker group was, in general, more internally divided at the stage where these case vignettes were presented, while forensic psychologists/psychiatrists were more in agreement of SMD at this stage. The core profile of information used in the three case vignettes was also mirrored in general use in FPI praxis, where the client’s self-report and the clinician’s observations were considered the most useful types regardless of case context, while some information types (e.g., cognitive testing) were only useful sometimes (i.e., varied with case context). Forensic social workers requested the least number of information sources within the cases, while forensic psychologists requested the most, but the difference in absolute numbers was minor and could be affected by the professional group’s different assessment focus in FPIs due to guidelines. In conclusion, this study indicates how to increase the chances of more analytic processing within FPI praxis and indicate areas for future research to diminish the risk of bias within the complex decision-making of FPIs.

## Data Availability Statement

The raw data supporting the conclusions of this article will be made available by the authors, without undue reservation.

## Ethics Statement

The studies involving human participants were reviewed and approved by Regional Ethical Committee in Gothenburg; Dnr: 940.16. The patients/participants provided their written informed consent to participate in this study.

## Author Contributions

MK, PA, and A-SLB were responsible for the study design and for producing the stimulus material for the study. OS, MK, and A-SLB were responsible for the data collection. OS and LG were responsible for the literature review. LG, MK, and OS conducted the analyses. LG and MK wrote the first manuscript draft. All authors revised the manuscript and contributed to the text.

## Conflict of Interest

The authors declare that the research was conducted in the absence of any commercial or financial relationships that could be construed as a potential conflict of interest.

## Publisher’s Note

All claims expressed in this article are solely those of the authors and do not necessarily represent those of their affiliated organizations, or those of the publisher, the editors and the reviewers. Any product that may be evaluated in this article, or claim that may be made by its manufacturer, is not guaranteed or endorsed by the publisher.

## References

[1] Raharjanti NW Wiguna T Purwadianto A Soemantri D Bardosono S Poerwandari EK Clinical reasoning in forensic psychiatry: concepts, Processes Pitfalls. *Frontiers Psychiatry.* (2021) 12:691377. 10.3389/fpsyt.2021.691377 34421677 PMC8374734

[2] Dror I. Cognitive and human factors in expert decision making: six fallacies and the eight sources of bias. *Anal Chem.* (2020) 92:7998–8004. 10.1021/acs.analchem.0c00704 32508089

[3] St Evans JBT Stanovich KE. Dual-process theories of higher cognition: advancing the debate. *Psychol Sci.* (2013) 8:223–4. 10.1177/1745691612460685 26172965

[4] Neal TMS Hight M Howatt B Hamza C. The cognitive and social psychological bases of bias in forensic mental health judgments. In: Miller MK Bornstein BH editors. *Advances in Psychology and Law.* Vol. 3 (Berlin: Springer) (2018). p. 151–75. 10.1007/978-3-319-75859-6_5

[5] Evans JS Dual-processing accounts of reasoning, judgment, and social cognition. *Annu Rev Psychol*. (2008) 59:255–78. 10.1146/annurev.psych.59.103006.093629 18154502

[6] Tversky A Kahneman D. Rational choice and the framing of decisions. In: Hogarth RM Reder MW editors. *Rational Choice: The Contrast Between Economics and Psychology.* (Chicago, IL: University of Chicago Press) (1987). p. 67–94.

[7] Kahneman D Frederick S. A model of heuristic judgment. In: Holyoak KJ Morrison RG editors. *The Cambridge Handbook of Thinking and Reasoning.* Cambridge: Cambridge University Press (2005). p. 267–93.

[8] Kahneman D. *Thinking, Fast and Slow.* New York, NY: Farrar, Straus and Giroux (2011).

[9] Dror I Murrie D. A hierarchy of expert performance applied to forensic psychological assessments. *Psychol Public Policy Law.* (2018) 24:11–23. 10.1037/law0000140

[10] Neal TMS Brodsky SL. Forensic psychologists’ perceptions of bias and potential correction strategies in forensic mental health evaluations. *Psychol Public Policy Law.* (2016) 22:58–76. 10.1037/law0000077

[11] Croskerry P Singhal G Mamede S. Cognitive debiasing 2: impediments to and strategies for change. *Br Med J Qual Saf.* (2013) 22(Suppl. 2):ii65–72. 10.1136/bmjqs-2012-001713 23996094 PMC3786644

[12] Neal TMS Grisso T. The Cognitive underpinnings of bias in forensic mental health evaluations. *Psychol Public Policy Law.* (2014) 20:200–11.

[13] Zapf PA Kukucka J Kassin SM Dror IE. Cognitive bias in forensic mental health assessment: evaluator beliefs about its nature and scope. *Psychol Public Policy Law.* (2018) 24:1–10. 10.1037/law0000153

[14] Pronin E Lin DL Ross L. The bias blind spot: perceptions of bias in self versus other. *Pers Soc Psychol Bull.* (2002) 28:369–81. 10.1177/0146167202286008

[15] Commons ML Miller PM Gutheil TG. Expert witness perceptions of bias in experts. *J Am Acad Psychiatry Law.* (2004) 32:70–5. 15497632

[16] Dror I. Human expert performance in forensic decision making: seven different sources of bias. *Aust J Forensic Sci.* (2017) 49:541–7. 10.1080/00450618.2017.1281348

[17] Beckham J Annis L Gustafson D. Decision making and examiner bias in forensic expert recommendations for not guilty by reason of insanity. *Law Hum Behav.* (1989) 13:79–87. 10.1007/BF01056164

[18] Dror I. A hierarchy of expert performance. *J Appl Res Mem Cogn.* (2016) 5:121–7. 10.1016/j.jarmac.2016.03.001

[19] Bornstein RF. Evidence–based psychological assessment. *J Pers Assess.* (2017) 99:435–45. 10.1080/00223891.2016.1236343 27808560

[20] Bornstein RF Hopwood CJ. Evidence–based assessment of interpersonal dependency. *Prof Psychol Res Pr.* (2017) 48:251–8. 10.1037/pro0000036

[21] Ægisdóttir S White MJ Spengler PM Maugherman AS Anderson LA Cook RS The meta-analysis of clinical judgment project: fifty-six years of accumulated research on clinical versus statistical prediction. *Couns Psychol.* (2006) 34:341–82. 10.1177/0011000005285875

[22] SFS 1962:700. *Brottsbalk. Om Val av Påföljd.* Switzerland: SFS (1962).

[23] Svennerlind S. *Philosophical Motives for the Swedish Criminal Code of 1965, Philosophical Communications, Web Series, 42.* Gothenburg: University of Gothenburg (2009).

[24] SOU 2015:52 Rapport från Bergwallkommissionen: Betänkande av Bergwallkommissionen. Stockholm: Elanders Sverige AB (2015).

[25] Prop. 1990/91:58. *Psykiatrisk Tvångsvård.* (1990). Available online at: https://data.riksdagen.se/fil/D8F7BD1F-FB87-4BE3-A487-9D99484DD372 (accessed March 14, 2022).

[26] Bennet T Radovic S. On the abolition and re-introduction of legal insanity in Sweden. In: Moratti S Patterson D editors. *Legal Insanity and the Brain: Science, Law and European Courts.* (Portland, OR: Hart Publishing Ltd) (2016). p. 169–206.

[27] Rättsmedicinalverket. *Statistik.* Stockholm: The National Board of Forensic Medicine (2021).

[28] Dressing H Salize HJ. Forensic psychiatric assessment in European Union member states. *Acta Psychiatrica Scand.* (2006) 114:282–9. 10.1111/j.1600-0447.2006.00771.x 16968366

[29] Brodsky S Dvoskin J Neal T. Temptations for the expert witness. *J Am Acad Psychiatry Law.* (2017) 45:460–3. 29282237

[30] Grondahl P. Scandinavian forensic psychiatric practices – an overview and evaluation. *Nord J Psychiatry.* (2005) 59:92–102. 10.1080/08039480510022927 16195105

[31] Messina E Ferracuti S Nicolò G Ruggeri M Kooijmans T Meynen G. Forensic psychiatric evaluations of defendants: Italy and the Netherlands compared. *Int J Law Psychiatry.* (2019) 66:101473. 10.1016/j.ijlp.2019.101473 31706393

[32] Rättsmedicinalverket. *Handledning för Forensisk Socialutredning.* Stockholm: The National Board of Forensic Medicine (2019).

[33] Rättsmedicinalverket. *Handledning för Psykologutredning.* Stockholm: The National Board of Forensic Medicine (2019).

[34] Rättsmedicinalverket. *Handledning för Medicinsk-Psykiatrisk Utredning.* Stockholm: The National Board of Forensic Medicine (2019).

[35] Rättsmedicinalverket. *Handledning för Rättspsykiatriskt Utlåtande.* Stockholm: The National Board of Forensic Medicine (2019).

[36] Neal TMS. Discerning bias in forensic psychological reports in insanity cases. *Behav Sci Law.* (2018) 36:325–38. 10.1002/bsl.2346 29672912

[37] Zapf PA Dror IE. Understanding and mitigating bias in forensic evaluation: lessons from forensic science. *Int J Forensic Ment Health.* (2017) 16:227–38. 10.1080/14999013.2017.1317302

[38] Sturup J Sygel K Kristiansson M. *Rättspsykiatriska Bedömningar i Praktiken – Vinjettstudie Och Uppföljning av Över 2000 Fall.* Stockholm: RMV (2013).

[39] Sygel K Sturup J Fors U Edberg H Gavazzeni J Howner K The effect of gender on the outcome of forensic psychiatric assessment in Sweden: a case vignette study. *Crim Behav Ment Health.* (2017) 27:124–35. 26648167 10.1002/cbm.1987

[40] Svensson O Andiné P Bromander S Ask K Lindqvist-Bagge A-S Hildebrand Karlén M. The decision-making process in Swedish forensic psychiatric investigations. *Int J Psychiatry Law.* (2022) 80:101709. 34924110 10.1016/j.ijlp.2021.101709

[41] McHugh ML Interrater reliability: the kappa statistic. *Biochem Med.* (2012) 22:276–82. 10.11613/BM.2012.031 PMC3900052 23092060

[42] Scarpazza C Zampieri I Miolla A Melis G Pietrini P Sartori G. A multidisciplinary approach to insanity assessment as a way to reduce cognitive biases. *Forensic Sci Int.* (2021) 319:110652. 10.1016/j.forsciint.2020.110652 33360246

[43] Engel GL. The need for a new medical model: a challenge for biomedicine. *Science.* (1977) 196:129–36. 10.1126/science.847460 847460

[44] Rättsmedicinalverket. *Årsredovisning.* Stockholm: The National Board of Forensic Medicine (2020).

[45] Kassin S Dror I Kukucka J. The forensic confirmation bias: problems, perspectives, and proposed solutions. *J Appl Res Mem Cogn.* (2013) 2:42–52. 10.1016/j.jarmac.2013.01.001

